# Characteristics and repair outcome of patients with Vesicovaginal fistula managed in Jimma University teaching Hospital, Ethiopia

**DOI:** 10.1186/s12894-016-0152-8

**Published:** 2016-07-12

**Authors:** Demisew Anemu Sori, Ahadu Workineh Azale, Desta Hiko Gemeda

**Affiliations:** Jimma University College of Public Health and Medical Sciences, Jimma, Ethiopia

**Keywords:** Vesicovaginal fistula, Obstetric fistula, Surgical repair

## Abstract

**Background:**

In Ethiopia, about 9000 fistula cases are estimated to occur every year with an incidence of 2.2/1000 women. This study was aimed to determine obstetric fistula characteristics and surgical repair outcomes among patients with fistula surgical repair.

**Methods:**

A Hospital based cross sectional study design was conducted on all patients with Obstetric vesicovaginal Fistula, who were admitted to Gynecology ward, and had surgical repair from January 2011 to December 2014. Data was collected from patients’ chart, operation logbook and discharge logbook which were filled up from the entry of the patient to the hospital till her discharge. At discharge, a dye test was done to determine the outcome of repair.

**Results:**

One hundred sixty eight patients with obstetric vesicovaginal fistula were repaired during the study period. The age of the women ranged from 12 to 45 years with mean of 25 (±6) years and 10.1 % were younger than 18 years. Eighty percent of patients were laboring for two or more days, 46.4 % delivered abdominally (cesarean section 24.4 %, hysterectomy for uterine rupture 22 %), and 85.7 % ended up in stillbirth. Most patients (56 %) had mid-vaginal vesicovaginal fistula. Route of repair was vaginal among 95.8 % of patients, and spinal anesthesia was applied among 70.8 % of patients. Out of 93.4 % patients who had successful closure of their fistula, 84.5 % of patients had their fistula healed and continent, 8.9 % of them developed urinary incontinence while 6.5 % of fistula repair had failed at the time of discharge.

**Conclusions:**

Most fistula patients in this study are older than 18 years, referred from health centers either for cephalopelvic disproportion or obstructed labor after prolonged labor at home. In this study, Spinal anesthesia as well as vaginal route was widely employed and high success rates were achieved with surgical repair. Therefore, increasing access to comprehensive emergency obstetric and new born care is essential to minimize the delay contributing to perinatal mortality and obstetric fistula. In addition use of spinal anesthesia and vaginal route of repair is essential for the high success of repair outcome and low postoperative morbidities.

**Electronic supplementary material:**

The online version of this article (doi:10.1186/s12894-016-0152-8) contains supplementary material, which is available to authorized users.

## Background

Obstetric fistula is a significant cause of maternal morbidity (maternal near-miss) sustained mostly by teenage women due to prolonged obstructed labor, in the majority labor obstructed for three or more days and delivery ended up in stillbirth in 78–93 % [1–5]. Globally, the exact prevalence of obstetric fistula is not known, however, in 2006, the WHO estimated that more than 2 million young women throughout the world live with untreated fistula and between 50,000 and 100,000 new women are affected each year [6]. In Ethiopia, where the maternal mortality ratio is high (676 per 100,000 live births), the overall prevalence of obstetric fistula among women of reproductive age (15–49 years) was estimated at 2.2–7.3 per 1000 women [4], with a total of 142,387 fistula cases and 9000 new cases occurring a year [7–9].

Though Classifications of obstetric vesicovaginal fistula (VVF) vary, based on anatomic classification, midvaginal vesical fistula is the commonest. In low income countries where access to maternity care is restricted, fistulae are associated with a prolonged or obstructed labor, most commonly occurring when a baby’s head becomes lodged in the mother’s pelvis cutting off blood flow to the surrounding tissues. Prolonged obstruction can cause the tissues to necrotize leading to fistula formation [10–15]. Though most obstetric fistulas are from the natural course of obstructed labor, iatrogenic fistula at the time of obstetric surgery is also rising [16, 17].

Obstetric fistula is a devastating maternal morbidity which leaves a woman with uncontrollable leaking of urine and/or feces or both from her vagina. Untreated obstetric fistula leads to debilitating physical, health and social problems including divorce, isolation and stigma by their husband and families, and economic dependency [18, 19]. Although surgical repair is the main stay of treatment of obstetric fistula with closure rate of 85 to 95 % and severe stress incontinence rate of 15–20 %, the repair outcome will be affected by different factors like; type of fistula, experience of the surgeon, route of repair, postoperative care and type of anesthesia [8, 13, 14, 16, 18–22].

In Ethiopia there are few health facilities including Jimma University teaching Hospital (JUTH) performing obstetric fistula repair with limited capacity and few expertise. The Hospital has been performing surgical repair since the past four years and it is crucial to determine the clinical characteristics and surgical outcomes of obstetric fistula repair.

In this study we sought to establish the clinical characteristics and outcomes in patients undergoing fistula repair at our facility.

## Methods

A Hospital based cross sectional study design was conducted on all patients with Obstetric vesicovaginal Fistula using a retrospective review of charts, who were admitted to Gynecology ward, Fistula treatment center and had surgical repair from January 2011 to December 2014 and who meet the inclusion criteria. English version pretested data collection format was used to collect Data on Socio-demographic, obstetric variables and physical examination findings by trained gynecology and obstetrics residents and fistula surgeons from a patient chart, operation logbook and discharge logbook which were filled up from the entry of the patient to the hospital till her surgery, post-operative period until discharge. All patients with obstetric fistula (Additional file 1) stayed in the ward for 14 days (for patients with vesical fistula without urethral involvement) to 21 days (for patients with vesical fistula with urethral involvement) with an indwelling urinary catheter after surgery and were followed up daily till discharge. At discharge, a dye test was done to determine the outcome of repair as almost all women will not comply to the three months follow up if there is no problem. In this study the outcome variables were Successful repair, Successful repair with incontinence and failure or unsuccessful closure. Successful repair was considered when a woman is continent and dry following fistula surgery after 14–21 days before discharge. Successful repair with incontinence was considered when a woman is wet of urine on stress but had a negative dye test. Failure or unsuccessful closure was considered when a woman is wet of urine and had a positive dye test after 14–21 days of continuous bladder drainage following fistula repair.

The Collected data was entered in to Epidata version 3.1, cleaned and analyzed using SPSS version 20 and interpretation, discussion and recommendations were made based on the findings.

An official letter was obtained from the Institutional Review Board of Jimma University to conduct this research and get permission from the Hospital. After permission was obtained, data was collected from patients’ chart, operation logbook, and from discharge logbook which was filled up during the study period. The outcome of this study has been communicated to the Department of Obstetrics and Gynecology and to the Hospital.

## Results

Among the total 200 vesicovaginal fistula patients repaired from January 2011 to December 2014 in Jimma University Teaching Hospital, 32 were excluded as their data was incomplete; and data of the remaining 168 were analyzed.

The age of the patients ranged from 12 to 45 years with mean of 25 (±6) years and 17 (10.1 %) were younger than 18 years. Sixty six (39.3 %) were primipara, 102 (60.7 %) were multiparous of which 40 (23.8 %) were grand multipara. One hundred thirty three (79.2 %) patients were laboring for two or more days while 24 (14.3 %) were laboring for more than three days during the causative pregnancy. One hundred twenty one (72 %) of patients delivered in the health facility, and 144 (85.7 %) of deliveries ended up in still birth. Regarding the mode of delivery, 67 (39.9 %) of patients delivered vaginally which may be spontaneous or after prolonged labor at home, 78 (46.4 %) had abdominal delivery out of which 41 (24.4 %) and 37 (22 %) were managed by cesarean section and hysterectomy respectively. Three patients had uterine repair after uterine rupture.

Based on anatomic classification, 94 (56 %) had mid-vaginal vesicovaginal fistula followed by circumferential vesicovaginal fistula (where bladder neck is totally detached from the urethra in these cases) 38 (22.6 %), juxta cervical 25 (14.9 %), juxta urethral 4 patients. For the majority, repairs were approached vaginally, among 161 (95.8 %), and under spinal anesthesia, among 119 (70.8 %). Fourteen (8.3 %) patients had failed previous fistula repair (Table 1).

**Table 1:** Socio-demographic characteristics and obstetric variables of vesicovaginal fistula patients Repaired between 2011 and 2014

Variable		Number (*n* = 168)	Percent
Age in years	<18 years	17	10.1
	≥18 years	151	89.9
Parity	I	66	39.3
	II-IV	62	36.9
	≥V	40	23.8
Duration of labor	1 day	35	20.8
	2–3 days	109	64.9
	>3 days	24	14.3
Place of delivery	Health facility	121	72.0
	Home	47	28.0
Mode of delivery	Vaginal delivery	67	39.9
	Instrumental	23	13.7
	Cesarean Section	41	24.4
	Hysterectomy	37	22.0
Special consideration (Goh classification)	Previous repair	14	8.3
	Circumferential	38	22.6
	Ureteric fistula	2	1.2
	No special consideration	114	67.9
Surgery approach	abdominal	7	4.2
	Vaginal	161	95.8
Type of anesthesia	General Anesthesia	49	29.2
	Spinal Anesthesia	119	70.8
Duration of bladder catheter	10 days	2	1.2
	14 days	124	73.8
	21 days	42	25.0
Type of obstetric fistula (anatomic classification)	Mid-vaginal VVF	94	56.0
	Circumfrential VVF	38	22.6
	Juxta cervical VVF	25	14.9
	Ureteric fistula	2	1.2
	Juxta urethral VVF	4	2.4
	Other	5	2.9
Fetal outcome	Alive	24	14.3
	Still birth	144	85.7

**Fig. 1:**
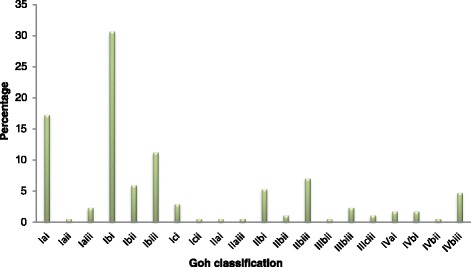
Goh classification of Vesicovaginal fistula patients repaired between 2011 and 2014

According to the Goh classification, most patients had simple fistula and were in the class Ibi among 30.7 % of patients followed by Iai among 17.3 % of patients, however 4.8 % of patients had IVbiii (Fig. 1).

Seventy eight (46.4 %) patients had operative abdominal delivery of which uterine rupture was the commonest indication among 40 (51.3 %) patients followed by obstructed labor among 35 (44.9 %) patients and fetopelvic disproportion among 3 patients. Fifteen out of 40 (37.5 %) patients presented with uterine rupture had bladder rupture and 4 (2.4 %) had iatrogenic bladder injury during cesarean section. Seven patients had neurologic injury at the time of admission and of which 6 patients had foot drop.

Regarding the postoperative complications, 16 (9.5 %) patients had urinary tract infection (cystitis and pyelonephritis) for which they were treated and get cured. Bladder was catheterized for 21 days for circumferential vesicovaginal fistulae, among 38 (22.8 %), and juxtaurethral fistulae, among 4 patients and for 14 days for midvaginal fistula, among 94 (56 %) patients. Out of 157 (93.4 %) patients who had successful closure of their fistula, 142 (84.5 %) had healed and continent, 15 (8.9 %) had developed urinary incontinence after their fistula was closed and healed while 11 (6.5 %) had failed fistula repair (Table 2).

**Table 2:** Obstetric variables and repair outcomes of vesicovaginal fistula patients repaired between 2011 and 2014

Variable		Number (*N* = 168)	Percentage
Bladder rupture	Yes	15	8.9
	No	153	91.1
Iatrogenic vesical fistula	Yes	4	2.4
	No	164	97.6
Postoperative complications	Anemia	3	15.8
	Pyelonephritis	4	21.1
	Cystitis	12	63.1
Indications for abdominal delivery	Fetopelvic disproportion	3	3.8
	Obstructed labor	35	44.9
	Uterine rupture	40	51.3
Neurologic injury	Foot drop	6	85.7
	Joint contracture	1	14.3
Fistula repair outcomes	Fistula healed and continent	142	84.5
	Fistula healed but incontinent	15	8.9
	Fistula not healed/failed	11	6.6

## Discussion

This study showed that most women with vesicovaginal fistula were older than 18 years. This finding is different from the previous studies where majority were teenage and predisposed to contracted pelvis and as a result obstructed labor which is the commonest cause of obstetric fistula in sub Saharan Africa [2, 3, 23]. Though the great proportion of patients gave birth for two or more times, more than a third gave birth for the first time. This might be explained by the fact that both primigravidity and multiparity were identified risk factors for obstetric fistula [20]. In this study, majority of patients had prolonged labor (80 %) which lasted for two or more days, delivered in the health facility (72 %), and about 86 % of deliveries were ended up in still births. These findings are similar with studies from sub-Saharan Africa [3, 4] except for the high rate of still birth in this study, which can be explained by the low health facility delivery rate of Ethiopia (10 %) [4], and in the current study, majority of patients were referred after prolonged labor either for uterine rupture or obstructed labor or feto-pelvic disproportion. These complications could have been averted by availing comprehensive emergency obstetric and new born care in each district [10, 24]. However, the still birth rate in the current study is lower than the previous study done in Ethiopia and Nigeria where the still birth rate is reported to be 93 % and 91.7 % respectively [3, 5].

From among forty six percent of abdominal deliveries, uterine rupture accounted for 23.8 %. This rate is very high when compared to other studies in Africa [20] and explained by the fact that most mothers had stayed laboring at home for long time and decided to seek health care late after complications has already developed [10, 11]. Fifty six percent of patients had mid-vaginal vesicovaginal fistula followed by circumferential vesicovaginal fistula and eight percent of patients had previous one fistula repair. These findings are similar with other studies done in Africa where 47 % of fistula was midvaginal [12, 14]. However, patients having previous failed repair are lower in this study compared to a study in Nigeria [16]. This may be explained by the fistula patients’ characteristics in the current study whereby most patients had simple fistula by Goh of Ibi (30.7 %) followed by Iai (17.3 %), though 4.8 % had IVbiii.

One aspect of surgical repair in particular, the route of repair undertaken, is critical as the abdominal (versus vaginal) approach may be associated with longer term hospitalization, Urinary tract infection (UTI) and increased blood loss [13, 21]. Type of anesthesia used is also an important factor which affects postoperative morbidities. In the current study, nearly 96 % had their repair transvaginally, 71 % under spinal anesthesia, when compared with most studies in other African countries [16]. Transvaginal route and spinal anesthesia is most widely used in the current study. This difference might be related to the background and experience of the fistula surgeon whereby some can perform almost all repairs vaginally including juxta cervical fistulas while others may prefer abdominal approach. However it is similar with a multistage study done in developing countries [14] where the vaginal approach accounts for 95.52 %. These might have contributed to the low postoperative morbidity (9.5 %) in the present study.

Nearly 38 % (15/40) of patients presented with uterine rupture had bladder rupture and only 4 patients had iatrogenic vesical fistula during cesarean section. In general, surgical skill of operating surgeon at the time of cesarean section is critical to avoid iatrogenic bladder fistula. The iatrogenic vesical fistula in this study is by far lower than the reports in most studies in Africa [16, 17]. Presence of neurologic injury mostly shows the severity of obstructed labor complex and may affect the repair outcome as well. In our study, the neurologic injury rate is 4 % which is very low when compared with a previous study done in East Africa [2, 17]. The difference can be explained by the fact that most patients in our study had simple fistula Goh Ibi followed by Iai which tells the degree of injury to the birth canal.

The overall fistula closure rate varies from center to center which may be affected by fistula characteristics and the experience of the surgeon. In the present study the overall closure rate was 93.4 % and 8.9 % of the patients developed urinary incontinence though their fistula was healed at discharge. This finding is comparable with studies in Africa [3, 17, 22] though there are reports of low fistula closure rate in another study [2, 20, 12].

In this study, some socio-demographic and Obstetric characteristics of fistula patients were missed because it was not filled up at the time of admission. In addition long term outcome was not assed because almost all patients would not come back for subsequent visits.

## Conclusions

Most fistula patients in this study are older than 18 years, referred from health centers either for cephalopelvic disproportion or obstructed labor after prolonged labor at home. In this study, Spinal anesthesia as well as vaginal route was widely employed and high success rates were achieved with surgical repair of vesicovaginal fistula. Therefore, increasing access to comprehensive emergency obstetric and new born care (CEmONC) at all levels of health system is essential. In addition use of spinal anesthesia and vaginal route of repair is essential for the high success of repair outcome and low postoperative morbidities.

## Abbreviations

CEmONC, comprehensive emergency obstetric and new born care; JUTH, Jimma University teaching Hospital; UTI, Urinary tract infection; VVF, vesicovaginal fistula

## Additional file

Additional file 1: Goh classification of Obstetric fistula. (DOCX 13 kb)
