# Supplementary material for: Characteristics and repair outcome of patients with Vesicovaginal fistula managed in Jimma University teaching Hospital, Ethiopia
Source: BMC Urol. 2016 Jul 12;16:41. doi: 10.1186/s12894-016-0152-8 (PMC4942998; doi:10.1186/s12894-016-0152-8)
Supplement: Additional file 1: — Goh classification of Obstetric fistula. (DOCX 13 kb) [file 12894_2016_152_MOESM1_ESM.docx]

**Goh classification of obstetric fistula (26)**

**Vesico-vaginal fistula**

Type 1: Distal edgeN3.5 cm from external urinary meatus

Type 2: Distal edge 2.5–3.5 cm from external urinary meatus

Type 3: Distal edge 1.5 tob2.5 cm from external urinary meatus

Type 4: Distal edgeb1.5 cm from external urinary meatus

(a) Sizeb1.5 cm in the largest diameter

(b) Size, 1.5–3 cm in the largest diameter

(c) SizeN3 cm in the largest diameter

i. None or only mild fibrosis (around the fistula and/or vagina), and/or vaginal lengthN6 cm with normal capacity

ii. Moderate or severe fibrosis (around the fistula and/or vagina), and/or reduced vaginal length and/or capacity

iii. Special consideration, e.g., post radiation, ureteric involvement, circumferential fistula, or previous repair

**Recto-vaginal fistulas**

Type 1: Distal edge of fistulaN3.5 cm from hymen

Type 2: Distal edge of fistulaN3.5 cm from hymen

Type 3: Distal edge of fistulaN3.5 cm from hymen

Type 4: Distal edge of fistulaN3.5 cm from hymen

(a) Sizeb1.5 cm in the largest diameter

(b) Size, 1.5–3 cm in the largest diameter

(c) SizeN3 cm in the largest diameter

i. No or mild fibrosis around the fistula and/or vagina

ii. Moderate or severe fibrosis

iii. Special consideration, e.g., post radiation, inflammatory disease, malignancy, or previous repair.
